# Mindful diagnostics: a central nervous system infection case study

**DOI:** 10.1017/ash.2026.10357

**Published:** 2026-04-10

**Authors:** Jonathan H. Ryder, Sarah E. Turbett

**Affiliations:** 1 Division of Infectious Diseases, Department of Internal Medicine, University of Nebraska Medical Center, Omaha, NE, USA; 2 Department of Pathology, Massachusetts General Hospital, Boston, MA, USA; 3 Division of Infectious Diseases, Department of Medicine, Massachusetts General Hospital, Boston, MA, USA; 4 Harvard Medical School, Boston, MA, USA

## Abstract

A clinical case is presented to discuss a framework for use of advanced diagnostics for central nervous system infections. Advantages, limitations, and diagnostic stewardship strategies are discussed for each modality: multiplex molecular meningitis/encephalitis panel, plasma microbial cell-free DNA sequencing, and cerebrospinal fluid metagenomic next generation sequencing.

## Introduction

Novel diagnostic methods play an important role in patients with central nervous system (CNS) infection. However, indiscriminate use of these assays is increasingly common, yet the vast majority of tests are negative, which can lead to misleading results with the potential for patient harm.^1–4^ Here, we use a clinical case to provide a framework for stewardship of CNS infection advanced diagnostics with a specific focus on the advantages, limitations, and best use of these tests.

## Clinical case

A male in his 50’s with a history of traumatic brain injury three months prior, requiring craniectomy followed by cranioplasty one month later, was admitted after a seizure resulting in a head strike. Computed tomography revealed a depressed cranioplasty flap with hardware loosening. Subsequently, he developed fevers; brain magnetic resonance imaging revealed a pseudomeningocele at the cranioplasty site. Cerebrospinal fluid (CSF) studies obtained via lumbar drain demonstrated total protein 198; glucose 28; 104,000 red blood cells; and 1,774 white blood cells with 86% neutrophils. Ceftriaxone, ampicillin, vancomycin, and acyclovir were initiated; CSF gram stain, bacterial culture, herpes simplex virus nucleic acid amplification test (NAAT), and blood cultures were unrevealing. Despite antimicrobials, fevers persisted; an infectious diseases specialist was engaged to determine the next best diagnostic steps. Specifically, the primary team asked about the utility of CSF multiplex molecular panel testing, plasma cell-free microbial DNA (cfDNA), and CSF metagenomic next general sequencing (mNGS).

## A diagnostic stewardship framework for CNS infection and potential stewardship applications

To determine the best diagnostic plan for this patient, we suggest the following three-step approach: (1) define the clinical syndrome and the goal of testing; (2) determine how the test result will change clinical management; and (3) understand the performance characteristics and operational fit of the test. In this case, the clinical syndrome is healthcare-associated bacterial meningitis/ventriculitis; the goal of testing is to identify the causative pathogen to optimize antimicrobial therapy and treatment duration. We describe the test performance and utility of multiplex molecular panel testing, plasma cfDNA, and CSF mNGS below; diagnostic stewardship strategies for implementing these tests are detailed in Table 1.

**Table 1.** Diagnostic stewardship interventions for central nervous system infection diagnostics

Diagnostic stewardship interventions for CNS infections
Create local algorithms and guidelines for judicial test utilization^11,14,15^
Restrict testing to expert consultants, eg, infectious disease and/or neurology^1,3,11^
Save excess initial CSF for CSF mNGS if needed at a later point, rather than sending CSF mNGS immediately^16^
Avoid use when low clinical suspicion for infection^11^
Require microbiology laboratory approval
Create clinical decision support that limits MEP testing to patients with confirmed CSF pleocytosis, immunocompromised status, or age <2 years^4,17^
Ensure timely antimicrobial stewardship program review of MEP panels to optimize antimicrobial use and test impact
Implement nudges in test comments, which emphasize risks of false positives/false negatives and utility of obtaining infectious diseases consultation for interpretation

CNS, central nervous system; CSF, cerebrospinal fluid; mNGS, metagenomic next generation sequencing; MEP, meningitis/encephalitis panel.

## CSF multiplex molecular panel testing

CSF multiplex molecular panel testing is a bundled set of NAATs for predefined pathogens associated with meningitis and/or encephalitis. As of December 2025, there is only one Food and Drug Administration (FDA) cleared and commercially available assay in the United States: the BioFire FilmArray Meningitis/Encephalitis Panel (MEP, bioMérieux, Salt Lake City, UT). This CSF assay rapidly detects 14 different targets with high analytical sensitivity and specificity (Table 2).^5^ Bacterial targets are aimed at pathogens that cause community acquired and neonatal meningitis with reports of high clinical sensitivity and specificity compared to traditional diagnostic methods, particularly in patients with antibiotic pretreatment.^6^ Importantly, correlation of MEP results with clinical features, CSF parameters, and other diagnostic testing is necessary to prevent misdiagnosis and unnecessary antimicrobial use given the potential for false positive results and detection of latent viruses. Additionally, although the MEP contains a broad range of community-acquired bacterial analytes, it does not contain targets for common causes of healthcare-associated bacterial meningitis/ventriculitis, including *Staphylococcus aureus,* coagulase-negative staphylococci, *Enterobacter* spp., *Pseudomonas* spp., *Acinetobacter* spp., and *Cutibacterium acnes,* limiting its utility when there is clinical concern for such a process, as in this case.^7^ Further advantages and limitations of MEP testing are described in Table 2.

**Table 2.** Test characteristics, advantages, limitations, and best uses of advanced diagnostic methods for meningitis/encephalitis

CSF multiplex molecular panel	Plasma cell-free microbial DNA	CSF metagenomic next generation sequencing
Specimen source		
CSF	Plasma	CSF

Technology		
Nested multiplex PCR	Metagenomic next generation sequencing	Metagenomic next generation sequencing

Targets		
Viruses: CMV, EV, HSV-1, HSV-2, VZV, HHV-6, HPeV Bacteria: *Escherichia. coli* K1, *Haemophilus influenzae, Listeria monocytogenes, Neisseria meningitidis, Streptococcus agalactiae, Streptococcus pneumoniae* *Yeast:* *Cryptococcus neoformans* ^*5*^	Over 1,000 DNA containing species^8^	Over 68,000 distinct species (DNA and RNA containing organisms)^12^

Turnaround time (TAT)		
∼1 hour from specimen receipt	∼1 day from specimen receipt^18^	1–2 days from specimen receipt^1^

Websites for information on specimen processing, transportation, and request forms		
Discuss with local or regional lab https://www.biomerieux.com/us/en/our-offer/clinical-products/biofire-meningitis-encephalitis-panel.html	https://kariusdx.com/our-solution/karius-spectrum	Delve Detect: https://www.delve.bio/product/ Mayo: https://www.mayocliniclabs.com/test-catalog/overview/620941

Advantages		
Broad range of pathogen detection for community-acquired meningitis and/or encephalitis Accurate results (98% sensitivity, >99% specificity) Rapid TAT Results less affected by antimicrobial administration compared to culture Relatively easy for the laboratory to perform and interpret results Can be performed in local or regional laboratory	Unbiased approach to diagnostic testing with broad range of DNA pathogen detection Can help identify rare pathogens and difficult to culture organisms Results less affected by antimicrobial administration compared to culture Noninvasive sampling method Reasonable TAT May be useful when CSF is unavailable	Unbiased approach to diagnostic testing with broad range of DNA and RNA pathogen detection Can help identify rare pathogens and difficult to culture organisms Results less affected by antimicrobial administration compared to culture Higher specificity for pathogen detection compared to plasma cell-free DNA

Limitations		
Limited pathogen detection for healthcare-associated meningitis and/or encephalitis Potential for false negative results (particularly for HSV and *Cryptococcus*) Potential for false positive results due to contamination (particularly *Streptococcus pneumoniae* and *S. agalactiae*), which can lead to excess antimicrobial use^19^ Can be challenging to interpret results of latent herpes viruses (eg, CMV, VZV, HHV-6) Does not replace traditional CSF culture, as does not provide antimicrobial susceptibility data or test for all potential organisms Higher cost compared to routine diagnostic methods (∼$200)^20^	Limited sensitivity: Negative result does not rule out infection if moderate-high clinical pretest probability Low microbial DNA concentrations in the blood compared to the CNS Only detects DNA containing organisms (several RNA organisms relevant for encephalitis, eg, WNV, Rabies, etc) Limited specificity: cannot distinguish between pathogenic and colonizing organisms Does not replace traditional CSF culture, as provides limited antimicrobial susceptibility data and does not test for all potential organisms High cost compared to routine diagnostic methods (∼$2,000)^3^ Only available in reference laboratories	Limited sensitivity Negative result does not rule out infection if moderate-high clinical pretest probability (WNV, *Borrelia burgdorferi* as examples) Limited specificity: cannot distinguish between pathogenic and colonizing organisms Does not replace traditional CSF culture, as does not provide antimicrobial susceptibility data or test for all potential organisms Slower TAT High cost compared to routine diagnostic methods (∼$3,000)^1^ Only available in reference laboratories

Best use case		
Can diagnose community-acquired bacterial or viral meningitis/encephalitis, particularly if antimicrobial pretreatment has already been initiated	Aid in the diagnosis of CNS infection from DNA containing pathogens when CSF or other invasive sampling is unavailable and standard diagnostic tests have not provided a diagnosis	Aid in the diagnosis of CNS infection when standard diagnostic tests have not provided a diagnosis or are unavailable

CSF, cerebrospinal fluid; CMV, cytomegalovirus; EV, enterovirus; HHV, human herpes virus; HPeV, human parechovirus; HSV, herpes simplex virus; PCR, polymerase chain reaction; TAT, turn-around time; VZV, Varicella zoster virus; WNV, West Nile Virus.

## Plasma cell-free microbial DNA

Microbial cfDNA testing relies on the isolation of microbial DNA fragments from the bloodstream. These fragments subsequently undergo NGS followed by identification by comparing the identified signatures to a large curated organism database.^8,9^ As of December 2025, Karius (Karius Inc., Redwood City, CA) is the only reference laboratory developed test that offers microbial cfDNA testing for commercial purposes.

As microbial cfDNA can circulate in the blood of healthy individuals, differentiating colonizing organisms from pathogens can be challenging. To help accomplish this, Karius classifies and quantifies any organism detected at or above their respective reportable thresholds.^8^ Organisms are classified as obligate pathogens (presence is likely to cause disease), commensal pathogens and DNA viruses (presence can represent disease or normal flora), or microbes with only published case reports (presence is unclear).^8^ Additionally, each identified organism is quantified using a standardized measurement unit (molecular per microliter) and for commensal pathogens and DNA viruses, a concentration gradient for each microbe is provided which is generated by comparing the identified concentration to those of the last 1,000 specimens that contained that target.^8^

Advantages and limitations of microbial cfDNA are described in Table 2. While microbial cfDNA has high analytical sensitivity and specificity, studies are lacking regarding the clinical utility of this technology for the diagnosis of meningitis/ventriculitis.^10^ One large retrospective study evaluated the clinical impact of microbial cfDNA testing; standardized chart review revealed most microbial cfDNA results (82%) did not change clinical management with the majority of results not being acted on by the clinical team.^2^ For meningoencephalitis specifically, no positive impact was seen in the 24 adult cases, suggesting limited utility of this testing strategy for this infectious syndrome. When possible, diagnostic testing directly from the site of infection (in this case CSF) is preferred to enhance both sensitivity and specificity to increase diagnostic accuracy. Microbial cfDNA can be considered when specimen testing in direct proximity to the site of infection is not feasible.

## CSF metagenomic next generation sequencing

CSF mNGS involves DNA and RNA extraction from CSF followed by NGS and bioinformatic processing to identify organisms from a predefined database. Organisms are reported if predefined total read thresholds were present after review by a clinical microbiologist.^1,11^ Currently, two laboratory developed CSF mNGS tests are commercially available in the US: Mayo CSF NGS (Mayo Clinic Laboratories, Rochester, MN) and Delve Detect (delvebio in partnership with University of California San Francisco, Boston, MA).^1,11^ Delve Detect utilizes a database of >68,000 microorganisms, while Mayo CSF NGS uses the National Center for Biotechnology Information database.^11,12^

Advantages and limitations of CSF mNGS are detailed in Table 2. While limited by sensitivity (63–70%), these tests have high specificity (93–99.6%).^1,11,13^ Factors associated with increased CSF mNGS test positivity include high pretest probability of CNS infection, immunocompromised status, antimicrobial administration prior to lumbar puncture, presence of CSF pleocytosis, and abnormal imaging.^1,11^ Significant limitations include potential for contaminants and false positives as well as identification of incidental organisms or those of unclear clinical significance.^1^ Contributing factors to false negative results include high host background DNA (eg, high pleocytosis), infections with low organismal burden at clinical presentation (eg, West Nile Virus, syphilis), and isolated CNS infection sites (eg, abscess or osteomyelitis).^1^

## Conclusion

In this case, CSF mNGS was performed and was positive for *Enterobacter cloacae* complex. Cefepime was initiated, and the patient clinically improved. This case illustrates that when used appropriately, advanced CNS diagnostics can improve diagnostic certainty and improve clinical care. By using the above framework combined with diagnostic stewardship interventions to promote “best-use” testing practices, diagnostic decisions can be optimized, ensuring diagnostic excellence for every patient.
